# Response of plant reflectance spectrum to simulated dust deposition and its estimation model

**DOI:** 10.1038/s41598-020-73006-2

**Published:** 2020-09-25

**Authors:** Jiyou Zhu, Xinna Zhang, Weijun He, Xuemei Yan, Qiang Yu, Chengyang Xu, Qun’ou Jiang, Huaguo Huang, Ruirui Wang

**Affiliations:** 1 Research Center for Urban Forestry of Beijing Forestry University, Key Laboratory for Forest Silviculture and Conservation of Ministry of Education, Key Laboratory for Silviculture and Forest Ecosystem Research in Arid- and Semi-Arid Region of State Forestry Administration, Beijing Forestry University, Beijing, 100083 China; 2 Research Institude of Tropical Forestry, Chinese Academy of Forestry, Guangzhou, 510520 Guangdong China; 3 Beijing Advanced Innovation Center for Tree Breeding By Molecular Design, National Engineering Laboratory for Tree Breeding, School of Nature Conservation, College of Biological Sciences and Technology, Beijing Forestry University, Beijing, 100083 China; 4 School of Soil and Water Conservation, Beijing Forestry University, Beijing, 100083 China

**Keywords:** Plant ecology, Plant physiology, Plant stress responses

## Abstract

To quantitatively reflect the relationship between dust and plant spectral reflectance. Dust from different sources in the city were selected to simulate the spectral characteristics of leaf dust. Taking *Euonymus japonicus* as the research object. Prediction model of leaf dust deposition was established based on spectral parameters. Results showed that among the three different dust pollutants, the reflection spectrum has 6 main reflection peaks and 7 main absorption valleys in 350–2500 nm. A steep reflection platform appears in the 692–763 nm band. In 760–1400 nm, the spectral reflectance gradually decreases with the increase of leaf dust coverage, and the variation range was coal dust > cement dust > pure soil dust. The spectral reflectance in 680–740 nm gradually decreases with the increase of leaf dust coverage. In the near infrared band, the fluctuation amplitude and slope of its first derivative spectrum gradually decrease with the increase of leaf dust. The biggest amplitude of variation was cement dust. With the increase of dust retention, the red edge position generally moves towards short wave direction, and the red edge slope generally decreases. The blue edge position moved to the short wave direction first and then to the long side direction, while the blue edge slope generally shows a decreasing trend. The yellow edge position moved to the long wave direction first and then to the short wave direction (coal dust, cement dust), and generally moved to the long side direction (pure soil dust). The yellow edge slope increases first and then decreases. The *R*^2^ values of the determination coefficients of the dust deposition prediction model have reached significant levels, which indicated that there was a relatively stable correlation between the spectral reflectance and dust deposition. The best prediction model of leaf dust deposition was leaf water content index model (y = 1.5019x − 1.4791, *R*^2^ = 0.7091, RMSE = 0.9725).

The problem of air pollution has always been one of the major topics of universal concern in the world^1,2^. In recent years, with the rapid development of China's transportation and industry and the influence of factors such as heating and coal burning in North China, air pollution generally shows a trend of continuous deterioration^3–5^. Especially in the Beijing–Tianjin–Hebei region of China, smog and dust weather are frequent, and air pollution has become one of the most serious environmental problems in the region, which has attracted extensive attention from the government and many researchers^5,6^. According to the data from Beijing Environmental Monitoring Center, the main pollutant in Beijing's atmosphere is inhalable particulate matter^7^. In order to further strengthen dust control, the Beijing municipal government set up a monitoring network of coarse particulate matter covering all streets and villages in the city in 2018^8^. Dust particulate matter not only seriously endangers the physical and mental health of urban residents, but also restricts the normal growth of urban vegetation^9–11^. With the worsening air quality, the impact of foliar dust on plants has become a research focus for researchers in the fields of environment, forest management and plant physiology at home and abroad.

Although the technology and methods of atmospheric quality monitoring have become increasingly mature at the present stage, including positioning monitoring stations, multi-dimensional monitoring of unmanned aerial vehicles, on-board monitoring and other methods^12,13^. The current monitoring methods generally cover a smaller range and are mostly point-to-area with greater time and space limitations as an important component of the city, urban greening vegetation has the characteristics of wide area and uniform distribution^13^. It not only beautifies the environment and maintains the ecological balance, but also plays an important ecological role in purifying the atmosphere. It is an important guarantee for the sustainable development of the city^14–18^. Urban greening plants can purify the environment by fixing and detaining atmospheric dust. However, dust cover will also cause adverse effects on urban plants^19–21^. In recent years, the research on the relationship between dust pollution and plant growth has mainly focused on the impact of leaf dust retention on plant phenotypic structure, the impact of leaf dust on plant communities and the physiological and ecological response of plant leaf dust retention^22–26^. Studies have shown that the dust retention capacity of plant leaves is often related to their own growth factors such as crown size, leaf number, total leaf area, leaf surface morphology and structure, and environmental factors such as atmospheric pressure, atmospheric temperature, precipitation, etc.^27–30^. In addition, researchers pointed out that dust particles covering the leaf surface not only affect the normal operation of plant photosynthesis, transpiration and respiration, but also lead to the disturbance of plant metabolism and normal growth and development, thus shortening the growth cycle of plants^31–35^. At the same time, the surface roughness of plant leaves is increased due to dust cover, which will also affect the spectral reflection of leaves to a certain extent^36,37^. The correlation between foliar dust and plant reflectance spectra, physiological ecology, etc. should be regarded as an important component of forest management and forest ecological function research, and more systematic and comprehensive research is urgently needed^38–40^.

In recent years, with the development of hyperspectral technology, the acquisition of ground hyperspectral data has become faster and more convenient^41^. Studying the model of retrieving the dust retention amount of blades from hyperspectral data can improve the monitoring efficiency of atmospheric dust fall and the density of spatial sampling points^42^. It can be used as an effective supplement to traditional monitoring methods of atmospheric dust fall, thus improving the time accuracy and spatial accuracy of atmospheric dust fall monitoring^43,44^. Based on this, many scholars have carried out relevant research on the spectral characteristics of dust retention in leaves. Reviewing the previous literatures, we found that most of the urban road environments studied were sampling points, which could not completely avoid the influence of environmental conditions such as light, water and temperature. Therefore, through indoor simulation experiments, the relative consistency of the growth characteristics and environmental impact of the experimental samples is ensured, and the errors caused by experimental operation are reduced. *Euonymus japonicus* is one of the most common urban greening tree species in China, which plays a vital role in urban landscape greening and air purification. More importantly, Euonymus japonicus is the broadleaf shrub with the largest planting area in Beijing. It plays a major role in dust retention in winter. Therefore, the study of dust retention ability of *Euonymus japonicus* can provide reliable basis for the analysis of atmospheric particulate pollution in Beijing.

In this study, *Euonymus japonicus* was taken as the research object, leaves with relatively consistent growth conditions were collected. Cement dust, coal dust and pure soil dust were collected, dust pollution simulation experiments were carried out indoors. Hyperspectral characteristics of *Euonymus japonicus* leaves under clean and dust pollution conditions were compared. We discussed the influence of dust pollution on the leaf surface reflectance spectrum of *Euonymus japonicus*, and established a prediction model of leaf dust retention based on spectral parameters. This provides a theoretical reference for the rational allocation of urban greening plants.

## Results

### Leaf reflectance spectral characteristics of different dust sources

**Figure 1:**
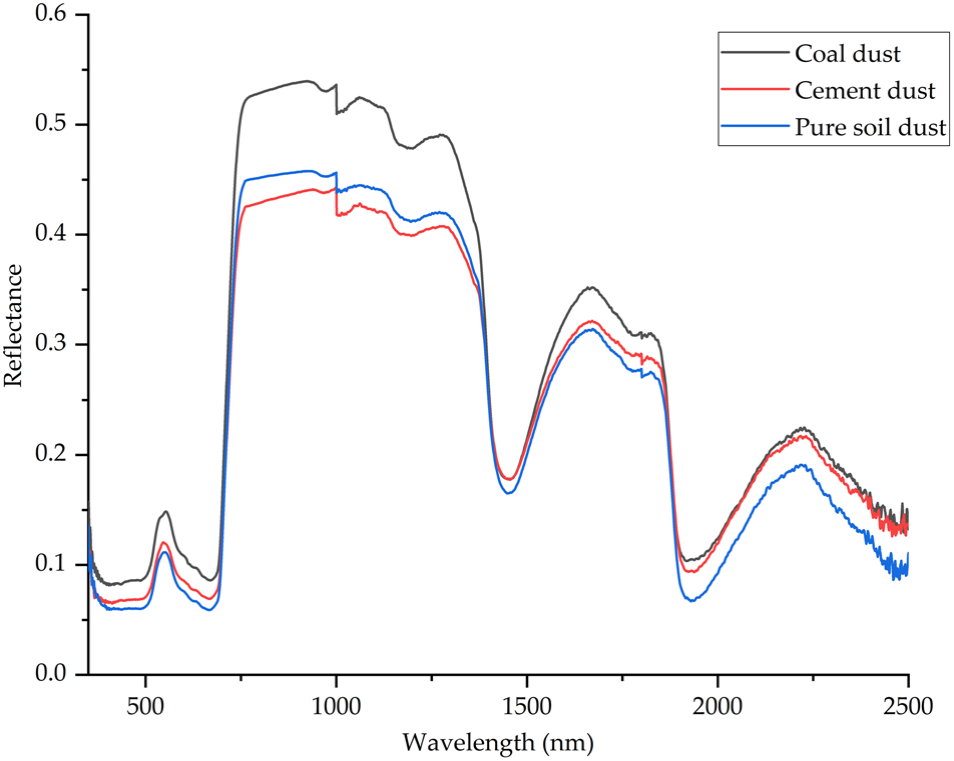
Leaf reflectance spectral curves.

The leaf surface reflectance spectrum of *Euonymus japonicus* has the following characteristics under different dust particles (Fig. 1). From visible light to near infrared band (400–2500 nm), there were 6 main reflection peaks and 7 main absorption valleys. The reflection peaks were 557 nm, 780 nm, 1000 nm, 1282 nm, 1660 nm and 2215 nm respectively. The absorption valleys were respectively in the ranges of 380–500 nm, 590–698 nm, 950–978 nm, 1000–1020 nm, 1154–1230 nm, 1300–1600 nm and 1848–2100 nm. Between 670–740 nm, 1451–1670 nm and 1928–2238 nm bands, the slope and reflectivity of the spectral curve vary greatly. This may be due to the influence of leaf cell structure, leaf surface moisture or leaf surface pollutants, and the absorption or transmission of spectrum by leaves, resulting in 6 high reflection peaks and 7 sharp drop reflection valleys^45,46^. In the visible light band (350–750 nm), the spectral reflectance was shown as coal dust > cement dust > pure soil dust. In the near infrared band (750–1100 nm), the spectral reflectance was shown as coal dust > pure soil dust > cement dust. It can be seen from this that this feature is sensitive to the pollution degree of the tree environment and the type of particulate matter.

### Effect of dust coverage on leaf reflectance spectrum

**Figure 2:**
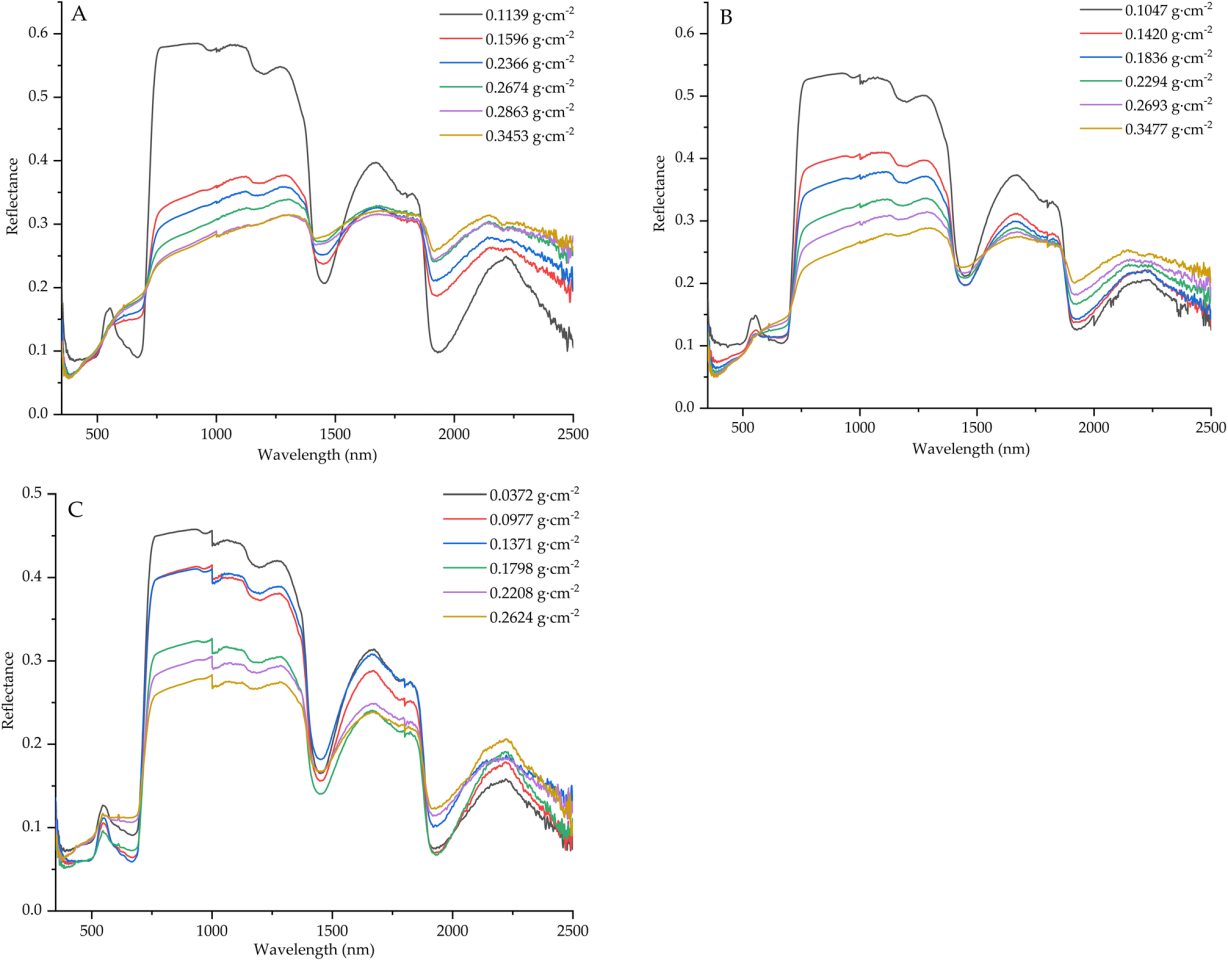
Foliar reflectance spectra under different dust coverage. (**A**) coal dust, (**B**) cement dust, (**C**) pure soil dust.

**Figure 3:**
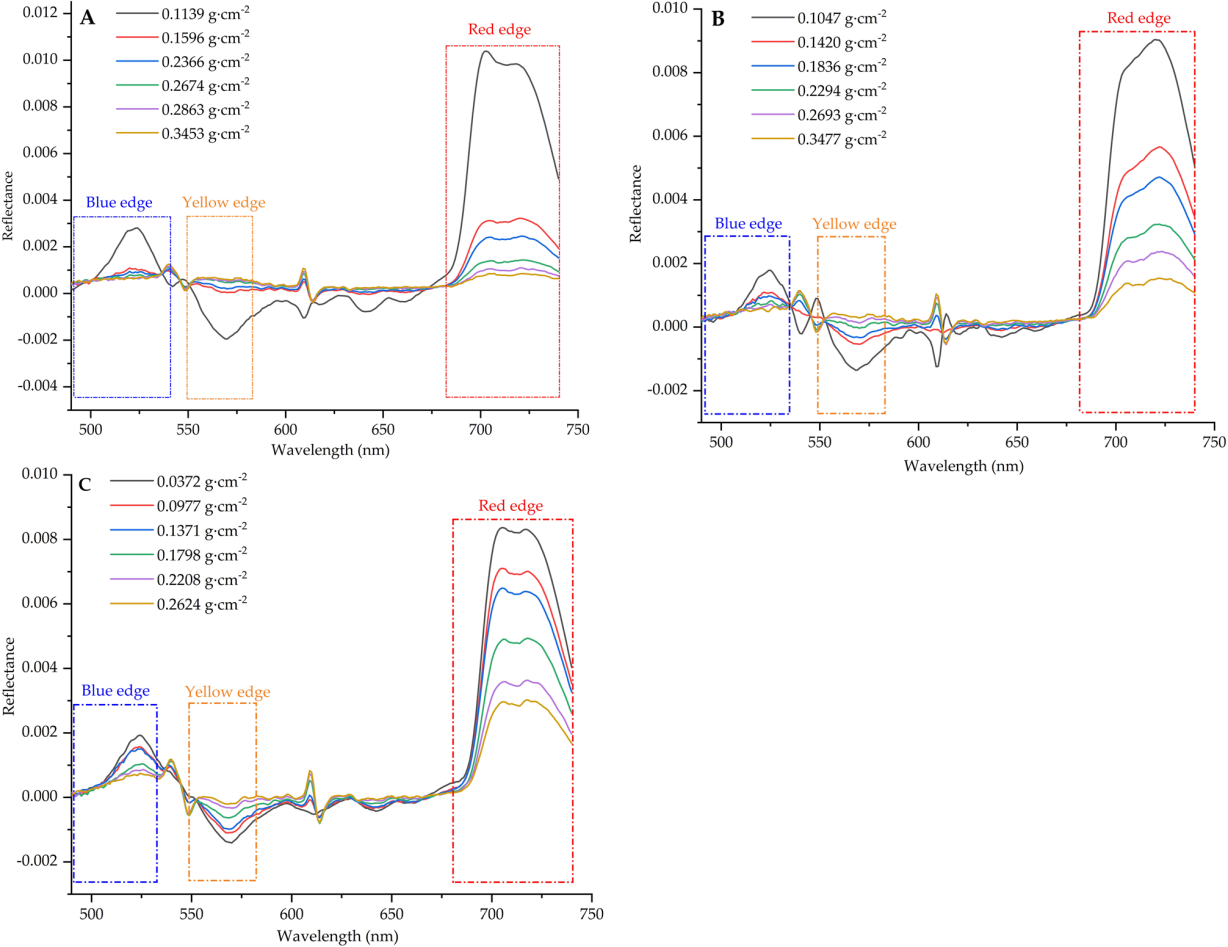
The first derivative spectral curves leaf. (**A**) coal dust, (**B**) cement dust, (**C**) pure soil dust.

As shown in Fig. 2, the spectral curves of black, red, blue, green, orange and purple were respectively the spectra after the dust was added for the first time to the sixth time (the same below). Figure 2A showed coal soil dust, and the corresponding dust amounts were 0.1139, 0.1596, 0.2366, 0.2674, 0.2863 and 0.3453 g m^−2^ in sequence. Figure 2B showed cement dust, with dust amounts of 0.1047, 0.1420, 0.1836, 0.2294, 0.2693 and 0.3477 g m^−2^ respectively. Figure 2C was pure soil dust, and the corresponding dust amounts were 0.0372, 0.0977, 0.1371, 0.1798, 0.2208 and 0.2624 g m^−2^ in sequence. As can be seen from Fig. 3, although the dust components were different, the spectral laws reflected by the three graphs were basically the same. When the amount of dust on the leaf surface per unit area increases gradually, the reflection spectrum trends were basically the same, and a steep reflection platform appears in the 692–763 nm band. In the range of 760–1400 nm, the spectral reflectance of leaf surface gradually decreases with the increase of dust coverage on leaf surface. On the contrary, in the range of 1900–2500 nm, the leaf surface reflectance shows a decreasing trend with the increase of dust amount, and the decreasing ranges are coal dust > cement dust > pure soil dust respectively.

### First derivative spectral characteristics of leaves with different dust coverage

**Figure 4:**
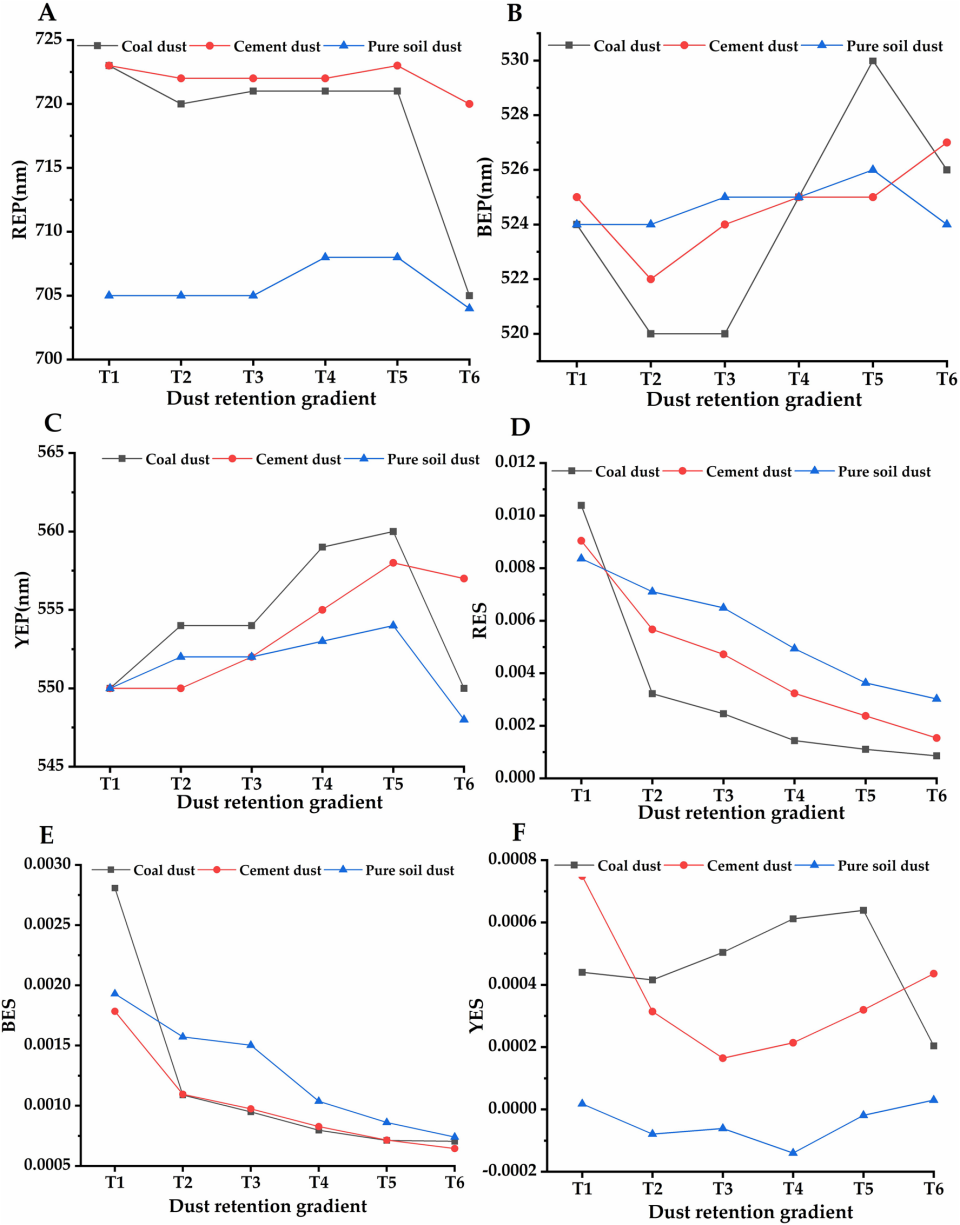
Three edge parameters of leaf spectrum under the influence of different dust retention amounts. (**A**) red edge position, (**B**) blue edge position, (**C**) yellow edge position, (**D**) red edge slope, (**E**) blue edge slope, (**F**) yellow edge slope. T1–T6 represent different dust retention amounts (Coal dust: T1-0.1139, T2-0.1596, T3-0.2366, T4-0.2674, T5-0.2863, T6-0.3453 g m^−2^; Cement dust: T1-0.1047, T2-0.1420, T3-0.1836, T4-0.2294, T5-0.2693, T6-0.3477 g m^−2^; Pure soil dust: T1-0.0372, T2-0.0977, T3-0.1371, T4-0.1798, T5-0.2208, T6-0.2624 g m^−2^).

The red edge slope is the point where the spectral reflectance growth rate reaches the maximum in the red edge region (680–750 nm). It is also the important inflection point and the most significant sign of the first derivative curve in the red edge region^47,48^. Red edge position is extremely sensitive to changes in chlorophyll content and internal cell structure, which is widely used in the calculation of plant yield, green amount and photosynthetic capacity, and the slope of red edge is mainly positively correlated with vegetation coverage and leaf area index^48^. The blue edge is the position where the first derivative of reflectivity reaches the maximum in 490–530 nm. The yellow edge is the minimum position of the first derivative of the reflectance of yellow light in the range of 550–582 nm^49,50^. As can be seen from Fig. 3, the spectral reflection in the red edge region of *Euonymus japonicus* leaves was quite different. In the near infrared region, the fluctuation amplitude and slope of its first derivative spectrum gradually decrease with the increase of the amount of leaf dust, of which cement dust has the largest variation amplitude. In the range of 680–740 nm, the spectral reflectance gradually decreases with the increase of leaf dust coverage. At the same time, although the first derivative curve of leaf surface spectrum of *Euonymus japonicus* showed basically the same general trend in different dust particles, there were two obvious reflection peaks in the 535–550 nm range of cement dust. This may be related to the reflection of specific particles in this band. Studies have shown that red edge was an indicator of vegetation nutrition, growth, water content, leaf area, etc., and has been widely used and confirmed^51,52^. When the vegetation biomass is large, the pigment content is high, and the growth is vigorous, the red edge position will move to the long wave direction^53,54^. However, when diseases and insect pests, pollution, leaf aging and other factors occur, the red edge position will move to the short wave direction^55^. In this study, we found that under the condition of different dust coverage, the red edge position generally moves towards short wave direction. With the increase of dust retention, coal dust has the greatest influence on the red edge position. The red edge slope generally decreases with the increase of dust retention. The blue edge position first moved towards the short wave direction and then moved towards the long side with the increase of dust amount, while the blue edge slope generally showed a decreasing trend with the increase of dust retention amount. The yellow edge moves to the long wave direction first and then to the short wave direction (coal dust, cement dust), and generally moves to the long side direction (pure soil dust) with the increase of dust retention. The yellow edge slope generally increases first and then decreases with the increase of dust retention (Fig. 4).

### Correlation analysis between dust retention amount of leaves and spectral single band

**Figure 5:**
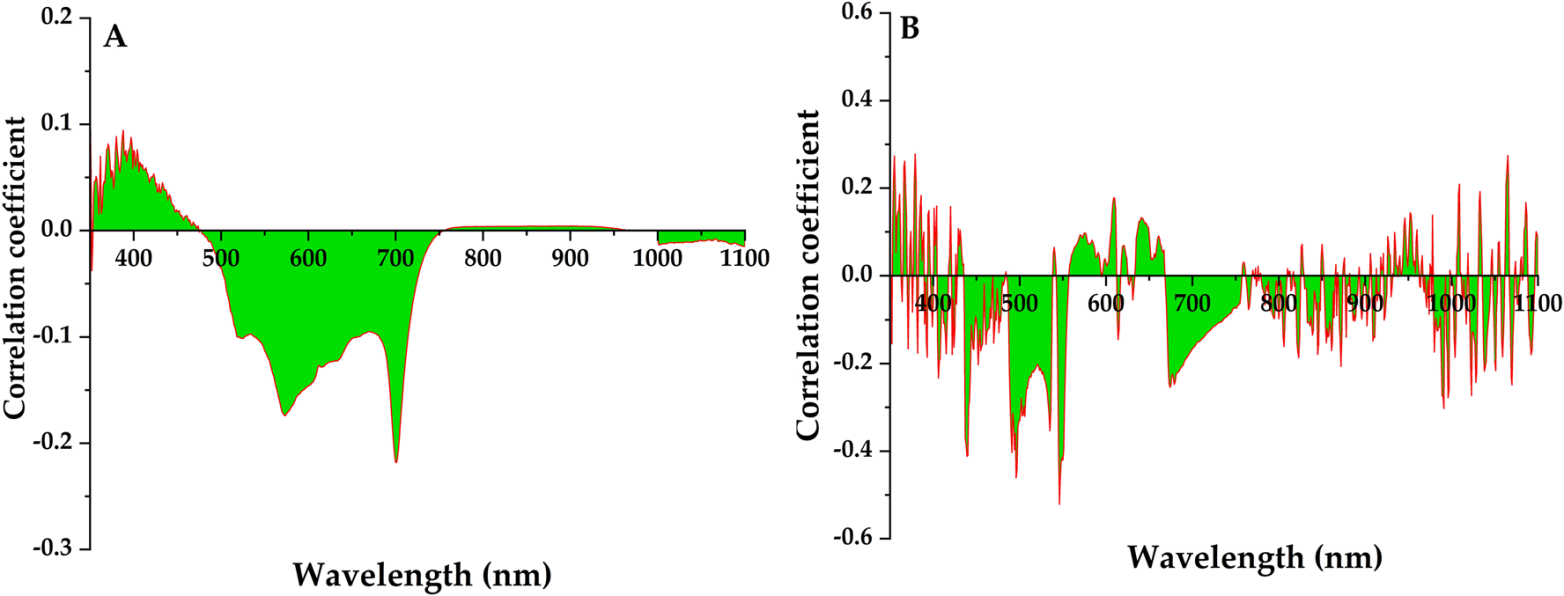
Analysis of relationship between dust weight and spectral reflectance. (**A**) Correlation between original reflectance and dust retention on leaf surface. (**B**) Correlation between first derivative reflectance and leaf dust retention.

The correlation between the amount of dust on leaf surface and spectral reflectance was calculated using Matlab software platform, with 50 samples in total. As shown in Fig. 5A, there was generally a negative correlation between the amount of dust on the leaves and the initial spectral information of the leaves. 490–750 nm and 1000–1100 nm were the main valley regions. There were three main peaks in these bands, which were 574 nm (− 0.1736), 701 nm (− 0.2182) and 1104 nm (− 0.0162). The dust retention of the blades in the range of 350–490 nm and 750–1000 nm was positively correlated with the spectral information, and the peak value appears at 350 nm (0.1298). As shown in Fig. 5B, there was a negative correlation between the amount of dust on the leaves and the spectral information of the first derivative of the leaves. The main valley values were in the range of 430–470 nm, 470–550 nm, 670–750 nm, 780–930 nm, 950–1100 nm. There were 6 main peaks in these bands, which were 431 nm (− 0.1041), 496 nm (− 0.4613), 546 nm (− 0.5218), 674 nm (− 0.2544), 872 nm (− 0.2065) and 991 nm (− 0.3026). The band positions with higher correlation were mainly located in the red edge (680–750 nm) and yellow edge (560–640 nm). This was because trilateral parameters, as the relevant parameters of spectral location characteristics, not only can reflect the spectral characteristics of vegetation well, but also are sensitive to changes in biochemical parameters of vegetation such as chlorophyll and water content^54–56^. As the red edge is closely related to various physical and chemical parameters of vegetation, it is an important indicator band to describe the state and health of plant pigment^55^. Dust falling on leaves was solid particles such as soil and construction dust, which were significantly lower than the reflectance of green vegetation in the near infrared band, thus causing the spectral reflectance of leaves with dust to decrease in this band, and this effect will increase with the increase of dust retention amount of leaves^56^.

### Establishment and screening of prediction model for dust retention in leaves

**Figure 6:**
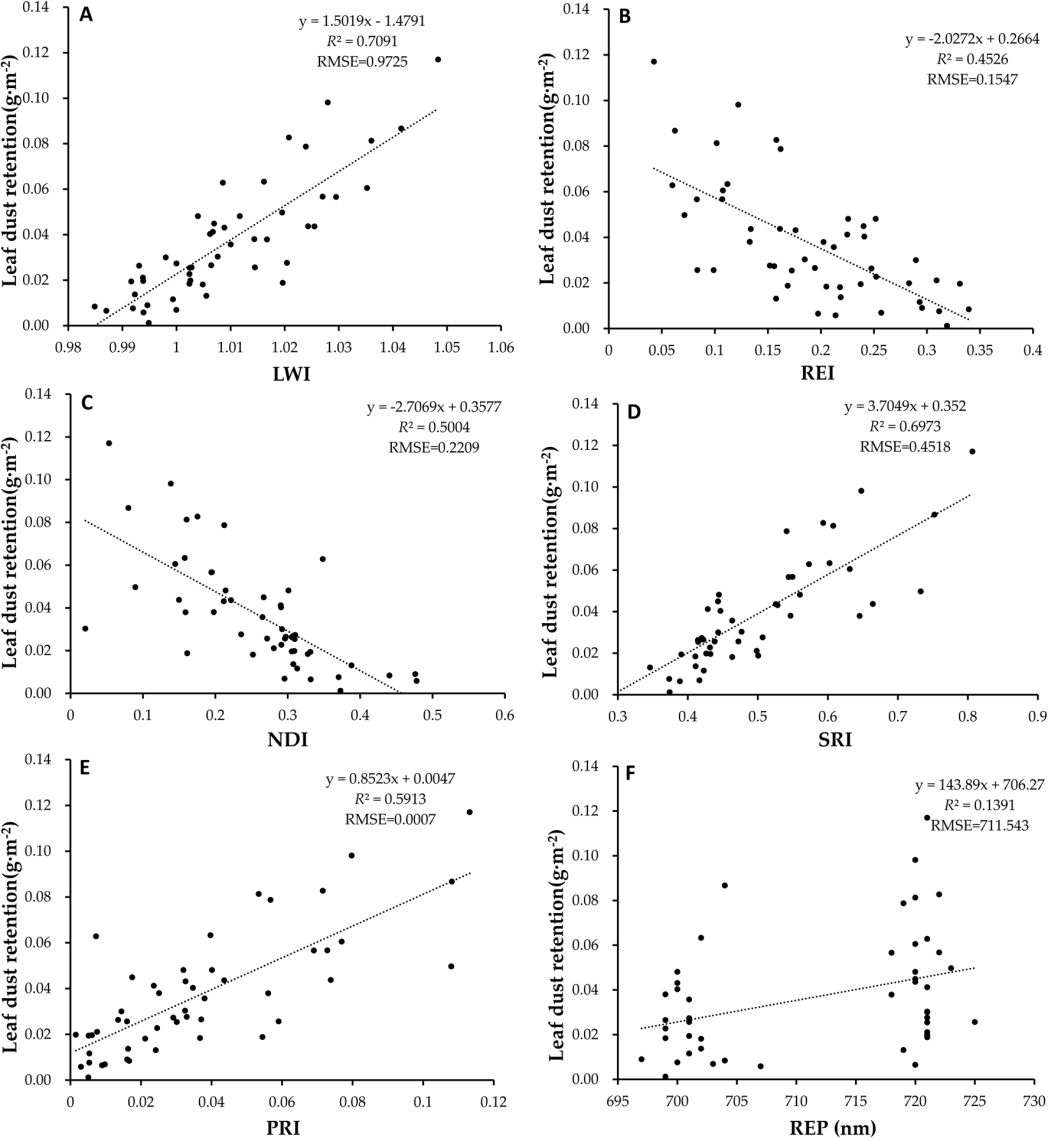
The parameter of spectral models for leaf dust content. (**A**) leaf water content index, (**B**) red edge index, (**C**) normalized index, (**D**) simple ratio index, (**E**) photosynthetic reflex index, (**F**) red edge position.

**Figure 7:**
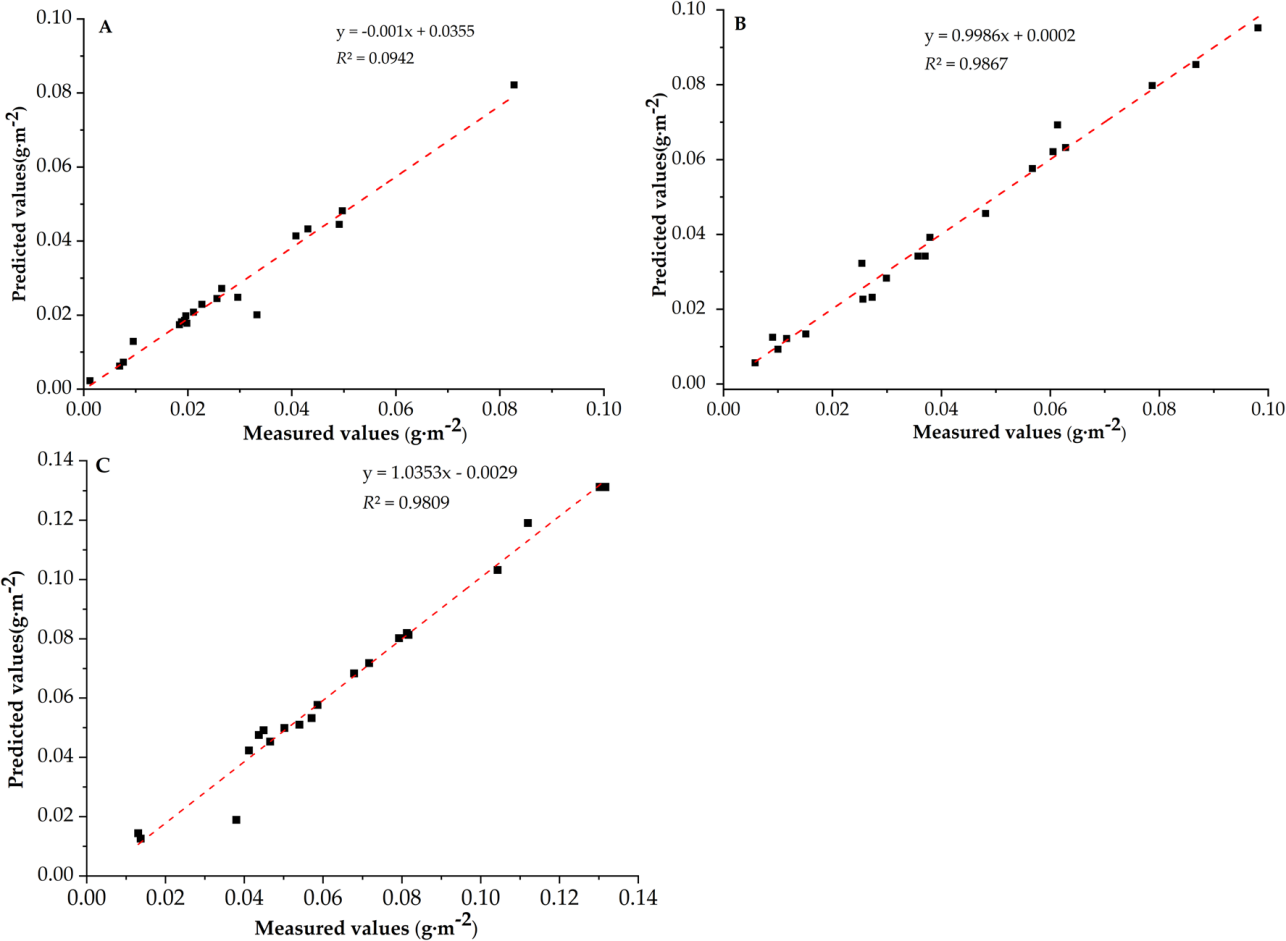
prediction model test. (**A**) coal dust, (**B**) cement dust, (**C**) pure soil dust.

Among many spectral parameters, spectral variables based on vegetation index (leaf water content index, red edge index, normalized index, simple ratio index and photosynthetic reflectance index) and variables based on spectral position (red edge, yellow edge and blue edge) were commonly used to reflect leaf spectral reflectance (Table 1)^57–59^. According to the correlation analysis between the spectral parameters and the amount of dust on the leaf surface (Table 2), the leaf surface water content index, red edge index, normalized index, simple ratio index, photosynthetic reflection index are highly significantly correlated with the amount of dust on the leaf surface, and the red edge position is significantly correlated with the amount of dust on the leaf surface (Fig. 6). Based on this, the above six spectral parameters were used as independent variables, and the leaf dust amount of *Euonymus japonicus* was used as dependent variable for regression analysis to establish linear prediction models of leaf dust amount respectively. Based on the above spectral parameters, 120 leaf samples were randomly selected to establish a prediction model of leaf dust retention of *Euonymus japonicus*. The results showed that the determination coefficient *R*^2^ values of the spectral prediction model for dust retention on leaf surface were leaf surface water content index (*R*^2^ = 0.7091), simple ratio index (*R*^2^ = 0.6973), photosynthetic reflectance index (*R*^2^ = 0.5913), normalized index (*R*^2^ = 0.5004), red edge index (*R*^2^ = 0.4526) and red edge position (*R*^2^ = 0.1391), respectively. The determination coefficient *R*^2^ values reached significant levels, indicating that there was a relatively stable correlation between spectral reflectance and dust retention. At the same time, their RMSE was relatively small, which indicated that the prediction model of these 6 parameters has high stability (Fig. 7). Therefore, the prediction model based on leaf water content index (y = 1.5019x − 1.4791, *R*^2^ = 0.7091, RMSE = 0.9725) has the best effect according to the principle of maximizing the determination coefficient *R*^2^. As shown in Fig. 4, the predicted value and the measured value based on the model were uniformly distributed above and below the standard baseline and were close to the baseline. At the same time, the correlation coefficients between predicted and measured values of pulverized coal, cement dust and pure soil dust are 0.9686, 0.9867 and 0.9809 respectively, and the root mean square error were 0.0013, 0.0001 and 0.0006 respectively. The prediction accuracy were 90.43%, 92.58% and 94.25% respectively.

**Table 1:** Definitions of spectral parameters.

Spectral parameters	Definition
**Variables based on vegetation index**	
Leaf water content index, LWI	R_970_/R_900_
Red edge index, REI	Sum of First Derivative of 680–750 nm Reflectance
Normalized index, NDI	(R_750_-R_705_ )/(R_750_ + R_705_ + 2R_445_ )
Simple ratio index, SRI	R_706_/R_809_
Photosynthetic reflex index, PRI	(R_570_-R_531_)/(R_570_ + R_531_)
**Variables based on spectral position**	
Red edge position, REP	RES wavelength position
Red edge slope, RES	Maximum first-order derivative value in red edge (680–750 nm)
Blue edge position, BEP	BES wavelength position
Blue edge slope, BES	Maximum first-order derivative value in blue edge (490–530 nm)
Yellow edge position, YEP	YES wavelength position
Yellow edge slope, YES	Maximum first-order derivative value in yellow edge (560–640 nm)

**Table 2:** Correlation analysis of spectral parameters and leaf dust retention based on Pearson analysis.

Spectral parameters	Correlation coefficient
LWI	0.8421**
REI	− 0.6728**
NDI	− 0.7074**
SRI	0.8350**
PRI	0.7689**
REP	0.3729*
RES	0.0899
BEP	0.2303
BES	− 0.1234
YEP	− 0.0470
YES	− 0.0104

## Conclusion

In order to quantitatively reflect the relationship between the intensity of dust pollution and the spectral reflectance of plants, this study selected the common sources of dust particles in cities (coal dust, cement dust and pure soil dust) to carry out the spectral measurement of plant dust on *Euonymus japonicus*. *Euonymus japonicu*s, as the winter evergreen broad-leaved tree with the largest distribution area in Beijing, plays an important role in atmospheric dust retention. Therefore, through indoor simulation experiments, the leaf spectral reflectance characteristics of *Euonymus japonicus* under the influence of different levels of dust pollution were analyzed, and the relationship between its spectral parameters and leaf dust retention was further explored, and the prediction model of leaf dust retention of *Euonymus japonicus* was established. The main conclusions were as follows.

1. Under the condition of different dust pollutants, the reflection spectrum of *Euonymus japonicus* leaves has 6 main reflection peaks and 7 main absorption valleys in the visible light to near infrared band (400–2500 nm), and the positions were basically the same.
2. When the amount of dust on the leaf surface per unit area increases gradually, the reflection spectrum trends were basically the same, and a steep reflection platform appears in the 692–763 nm band. In the range of 760–1400 nm, the spectral reflectance of leaf surface is negatively correlated with the dust coverage of leaf surface.
3. In the near infrared region, the fluctuation range and slope of the first derivative spectrum gradually decrease with the increase of the amount of foliar dust, with cement dust having the largest variation range. In the range of 680–740 nm, the spectral reflectance gradually decrease with the increase of leaf dust coverage. Under the condition of different dust coverage, the red edge position has not changed obviously, but its red edge slope and red edge index decrease continuously with the increase of dust content.
4. The red edge position generally moves toward short wave direction with the increase of dust retention, while red edge slope generally decreases with the increase of dust retention. Blue edge position first moved towards the short wave direction and then moved towards the long side with the increase of dust amount, while the blue edge slope generally showd a decreasing trend with the increase of dust retention amount. The yellow edge moved to the long wave direction first and then to the short wave direction (coal dust, cement dust), and generally moved to the long side direction (pure soil dust) with the increase of dust retention. On the whole, the slope of the yellow edge increases first and then decreases with the increase of dust retention. There were two obvious reflection peaks of cement dust in 535–550 nm, which may be related to the reflection of specific particles in this band.
5. The determination coefficient *R*^2^ values of the spectral prediction model of leaf dust retention were leaf water content index (*R*^2^ = 0.7091), simple ratio index (*R*^2^ = 0.6973), photosynthetic reflectance index (0.5913), normalized index (*R*^2^ = 0.5004), red edge index (*R*^2^ = 0.4526) and red edge position (0.1391) from large to small, respectively. The determination coefficient *R*^2^ values reach significant levels, indicating that there was a relatively stable correlation between spectral reflectance and dust retention. At the same time, their RMSE was relatively small, which indicates that the prediction model of these 6 parameters has high stability. The best prediction model of leaf dust retention was leaf water content index model (y = 1.5019 x − 1.4791, *R*^2^ = 0.7091, RMSE = 0.9725).

## Materials and methods

### Sample collection

**Figure 8:**
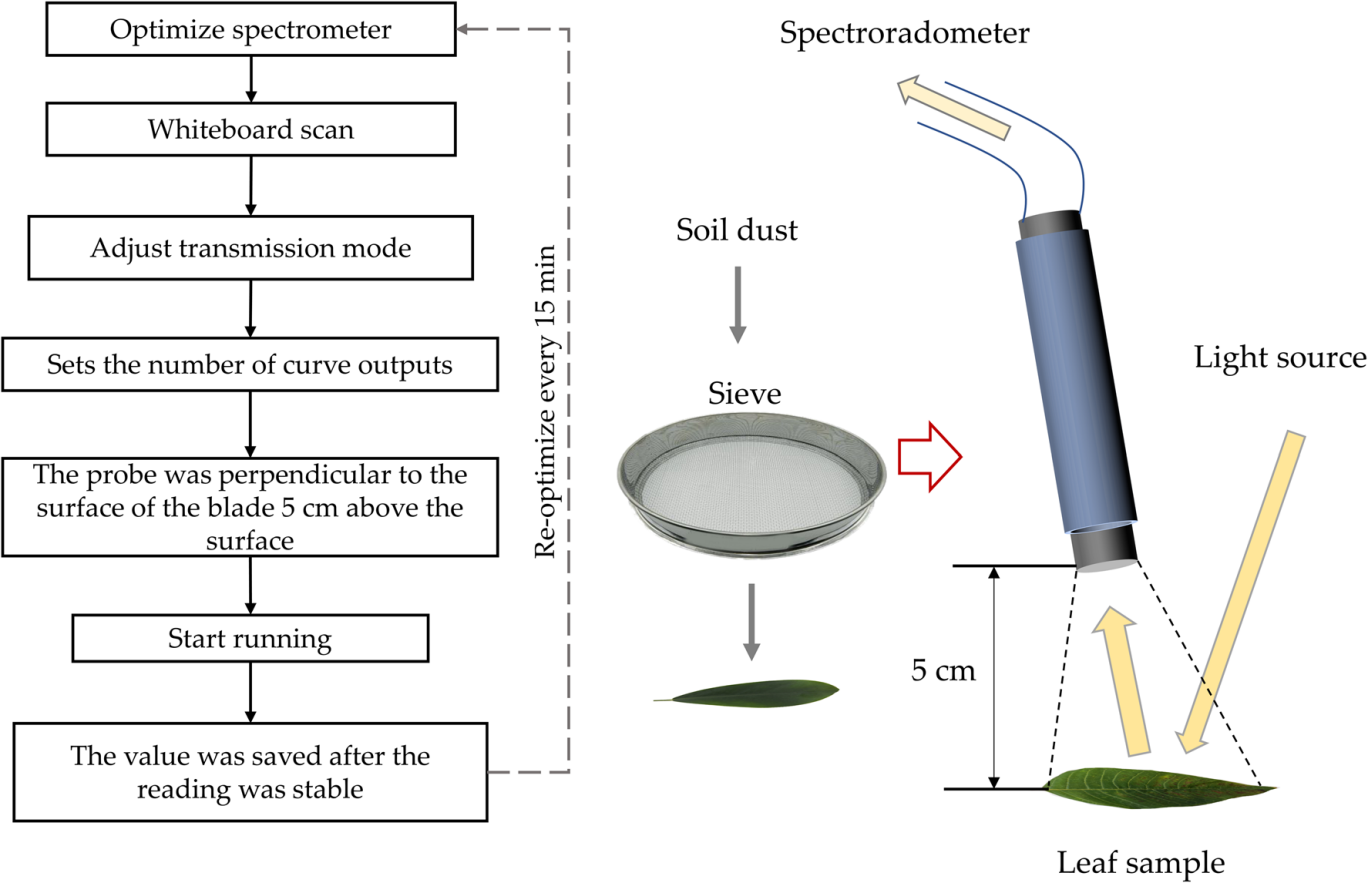
Leaf dust dust simulation and spectrometry demonstration.

In order to effectively use green plants to prevent and control atmospheric particulate pollution. It is necessary to combine plant retention and dust tolerance to find out the dust retention ability and stress resistance of plants under specific environment. Therefore, we collected dust from different urban environments as a source of test dust in this study. In October 2019, dust was collected in cement plants, coal mines and parks respectively and put into sealed bags for later use. At the same time, *Euonymus japonicus*, which was completely exposed to sunlight and has no high-rise shelter, was selected on the campus of Beijing Forestry University. The sampling time was sunny and windy 3 weeks before the sampling day. Randomly collected 180 mature, healthy and disease-free leaf samples, put them into a clean tray, and then take them back to the laboratory for measurement. From outdoor sample collection to indoor experiment simulation test, the interval time was controlled within 10 min to ensure that the sample was close to the natural growth state. The flow chart of leaf reflection spectrum measurement is shown in Fig. 8^60^.

### Dust pollution simulation and leaf surface spectral determination

First, the leaf area (LA, cm^2^) was measured in sequence by serial number using LI-3000C portable leaf area scanner (US, LI-COR). Then, after the collected soil sample is naturally air-dried, it is sieved to the surface of the corresponding leaf sample (the distance between the bottom of the sieve and the leaf surface is 20 cm) by a sieve with a pore diameter of 0.1 mm, and then it is moved to the analytical balance with sanitary sharp-nosed tweezers to weigh the dust (dust weight, g). The retention of dust on leaf surface per unit area is the ratio of mass difference to leaf area before and after dust increase. The steps of spectrum data acquisition are: weighing, spectrum measurement, dust removal, secondary weighing and secondary spectrum measurement. The weight of the blade was measured using an electronic balance of one in ten thousand. The spectrum was measured by FieldSpec3 near infrared spectrometer (Analytical Spectral Device, Almero, Netherlands), and the detection band range was 300–2500 nm. The sampling interval is 1.4 nm, the field angle is 30, and the resolution is 3–700 nm. The steps of spectrum data acquisition are as follows: dark scanning, white board scanning, adjusting transmission mode, measuring the probe 5 cm above the blade surface vertically, and saving the value after the reading is stable.

### Data processing

The spectral data were processed by View Spectral Pro 6.0 software, and the prediction model and plot were established in Excel 2019, Origin Pro2019b and Matlab and other data processing software.
